# Predictors of In-Hospital Mortality in Traumatic Acute Subdural Hematoma: The Role of Admission International Normalized Ratio, Imaging Parameters and Neurological Severity

**DOI:** 10.3390/biomedicines14061388

**Published:** 2026-06-19

**Authors:** Serban Iancu Papacocea, Miruna Ioana Lazăr, Romica Cergan, Ioana Anca Badarau, Toma Marius Papacocea

**Affiliations:** 1 Medical Physiology Department, Faculty of Medicine, “Carol Davila” University of Medicine and Pharmacy, 020021 Bucharest, Romania; serban-iancu.papacocea@drd.umfcd.ro (S.I.P.); anca.badarau@umfcd.ro (I.A.B.); 2 Neurosurgery Department, “Saint Pantelimon” Hospital, Faculty of Medicine, “Carol Davila” University of Medicine and Pharmacy, 020021 Bucharest, Romania; toma.papacocea@umfcd.ro; 3 Alergology and Clinical Immunology Department, “Nicolae Malaxa” Clinical Hospital, 022441 Bucharest, Romania; miruna-ioana.lazar@rez.umfcd.ro; 4 Anatomy Department, Faculty of Medicine, “Carol Davila” University of Medicine and Pharmacy, 020021 Bucharest, Romania

**Keywords:** subdural hematoma, traumatic brain injury, coagulation, Glasgow Coma Scale, international normalized ratio, midline shift, mortality, neurosurgery, prognostic factors

## Abstract

**Background/Objectives**: Acute subdural hematomas (aSDH) represent a frequent and potentially life-threatening form of traumatic intracranial hemorrhage. This study aims to assess prognostic factors associated with mortality and clinical outcome, with particular emphasis on coagulation-related parameters, especially international normalized ratio (INR). **Methods**: A single-center retrospective cohort study was performed. We included 151 patients with traumatic aSDH, admitted between January 2020 and June 2025 to the Department of Neurosurgery of the Clinical Emergency Hospital “Saint Pantelimon”. Demographic, clinical, laboratory, and imaging parameters obtained at admission were analyzed. Univariate and multivariable regression analyses were performed to identify predictors of in-hospital mortality. Internal validation included bootstrap resampling, calibration analysis, penalized regression and spline modeling. **Results**: The cohort had a mean age of 67.4 years and was predominantly male (72.8%). Overall, in-hospital mortality was 36.4%, while 58.3% of patients underwent surgical intervention. Admission Glasgow Coma Scale (GCS) score represented the strongest predictor of mortality. Hematoma thickness was significantly associated with midline shift, mortality, and surgical intervention. Elevated INR was significantly associated with increased hematoma thickness, greater midline shift, lower GCS, and increased mortality. In multivariable analysis, INR ≥ 1.4 remained independently associated with mortality (OR 4.08, 95% CI 1.56–11.29, *p* = 0.005), together with lower GCS. The final model demonstrated very good discrimination (AUC 0.887) and good calibration. **Conclusions**: Outcome in traumatic aSDH appears to be influenced by neurological severity, hematoma burden, and coagulation status. Admission GCS remained the strongest predictor of mortality, while elevated INR independently predicted poor outcome.

## 1. Introduction

ASDHs are hemorrhagic intracranial lesions in which blood rapidly accumulates between the arachnoid membrane and the cranial dura mater. They are almost invariably associated with traumatic brain injury (TBI) and are typically caused by the rupture of bridging veins (superficial cerebral veins that transport venous blood from the cerebrum towards the dural venous sinuses) following acceleration–deceleration mechanisms [1]. Rare cases of non-traumatic aSDHs have also been described, most frequently caused by hemostatic disturbances, such as anticoagulant overdose, disseminated intravascular coagulation or liver cirrhosis [2]. However, the present study focuses exclusively on traumatic aSDHs. Other forms of traumatic intracranial hemorrhagic lesions include epidural hematoma (between the inner skull table and the outer layer of the dura mater), traumatic subarachnoid hemorrhage (which should be differentiated from the subarachnoid hemorrhage caused by aneurysmal rupture) and cerebral contusion/traumatic intraparenchymal hemorrhage.

### 1.1. Epidemiology

TBI remains a major cause of mortality and long-term disability worldwide. Globally, TBI affects approximately 69 million individuals each year, with an annual reported incidence of 939 cases per 100,000 inhabitants [3]. Epidemiological patterns vary according to geographic region and socioeconomic status. As such, in developing countries, in which the population remains predominantly young and traffic regulations are often less stringent, the majority of TBIs occur as a result of road traffic accidents, whereas in developed countries, largely due to increasingly aging populations, accidental falls represent the leading cause of TBIs [3,4,5,6].

As far as severity is concerned, TBIs are classified as mild, moderate or severe, based on GCS. As such, we describe mild TBIs (GCS 13–15), moderate TBIs (GCS 9–12), and severe TBIs (GCS 3–8) [7].

### 1.2. Objectives

The increasing incidence of fall-related TBI among elderly patients has important clinical implications, particularly in the context of the widespread use of antithrombotic medication in this population. Elderly individuals frequently present with multiple cardiovascular comorbidities, requiring chronic antiplatelet or anticoagulant medication, for conditions such as atrial fibrillation, valvular heart disease, venous thromboembolism or post-stenting status [8]. Although these pharmaceutical agents play a fundamental role in thromboembolic prevention, they may also contribute to unfavorable outcomes following TBI by increasing the risk of hemorrhagic progression, impaired intraoperative hemostasis, prolonged operative time, and postoperative rebleeding. As a result, hemostatic disturbances have become increasingly relevant factors in the management and prognostic assessment of patients with aSDH. However, the prognostic significance of coagulation abnormalities in traumatic aSDH remains insufficiently characterized, particularly in relation to established clinical and radiological severity markers.

The present study aims to evaluate prognostic factors associated with in-hospital mortality in patients presenting with aSDH, with particular emphasis on coagulation-related parameters, especially international normalized ratio (INR), in correlation with clinical and radiological severity markers.

## 2. Materials and Methods

We performed a retrospective single-center cohort study including patients admitted between January 2020 and June 2025 to the Department of neurosurgery of Clinical University Hospital “Saint Pantelimon”, Bucharest.

Ethical approval for retrospective retrieval and analysis of patient data was obtained from the Ethics Committee of Clinical Emergency Hospital “Saint Pantelimon” (protocol no. 75289/29.07.2025). The present study forms part of a broader PhD research project that subsequently received approval from the Institutional Review Board of “Carol Davila” University of Medicine and Pharmacy (protocol no. 11706/08.05.2026).

Patients were identified through the hospital electronic registry using the ICD-10 diagnostic code S06.5 (traumatic subdural hemorrhage). A total of 151 patients with aSDH were included, regardless of their surgical status. The diagnosis was confirmed using non-contrast cranial computed tomography (CT). All laboratory, clinical and imaging parameters were obtained at admission. Data completeness was assessed prior to analysis. No missing values were identified for the variables included in the primary analyses and multivariable model; therefore, no imputation procedures were required. Variables with incomplete data (e.g., admission serum fibrinogen) were excluded from the corresponding analyses.

Inclusion criteria:

- Patients with aSDH on CT imaging, defined as a crescent-shaped hyperdense collection located along the lateral aspect of the inner table of the skull.
- Patients admitted to the Department of Neurosurgery for either surgical evacuation or conservative management and neurological monitoring.

Exclusion criteria:

- Associated intraparenchymal hematoma/contusion.
- Associated epidural hematoma.
- Penetrating cranial trauma.
- Bilateral hematomas.
- Chronic subdural hematomas.
- Interhemispheric (falcine) subdural hematoma.
- Tentorium cerebelli subdural hematoma.

These exclusion criteria were applied in order to obtain a more homogeneous cohort and reduce confounding generated by associated intracranial traumatic lesions.

Statistical analyses were performed using R software version 4.4.1 (R Foundation for Statistical Computing, Vienna, Austria), IBM SPSS Statistics version 26 (IBM Corp., Chicago, IL, USA), and GraphPad Prism version 8.0.2 (GraphPad Software, San Diego, CA, USA). Continuous variables were summarized as mean ± standard deviation or median (interquartile range), as appropriate, depending on data distribution (assessed by Shapiro–Wilk and Kolmogorov–Smirnov normality tests, as well as visual inspection of data distribution), whereas categorical variables were summarized as frequencies and percentages. Group comparisons were conducted using Mann–Whitney U test for continuous variables.

Multivariable logistic regression was used to identify independent predictors of in-hospital mortality. Predictor selection was based on clinical relevance and exploratory univariate analyses. The functional form of continuous predictors was assessed using restricted cubic splines to evaluate potential nonlinearity. Based on these analyses, INR was modeled as a categorical variable using a clinically interpretable threshold (≥1.4).

Model performance was evaluated in terms of discrimination, calibration, and clinical utility. Discrimination was assessed using the area under the receiver operating characteristic curve (AUC) with 95% confidence intervals estimated by the DeLong method. Calibration was evaluated using calibration plots, calibration intercept and slope, and overall prediction error metrics including the Brier score, mean absolute error, and mean squared error. Internal validation was performed using bootstrap resampling (1000 iterations) to obtain bias-corrected estimates. Decision curve analysis was conducted to assess net clinical benefit across a range of threshold probabilities.

To assess model stability and potential overfitting, penalized logistic regression using least absolute shrinkage and selection operator (LASSO) was performed, and model discrimination was compared with the standard model using DeLong’s test. Multicollinearity was evaluated using variance inflation factors (VIF).

Finally, causal mediation analysis was conducted to investigate whether hematoma thickness mediates the relationship between elevated INR and mortality. The average causal mediation effect (ACME), average direct effect (ADE), total effect, and proportion mediated were estimated using nonparametric bootstrap methods (1000 simulations).

A two-sided *p*-value < 0.05 was considered statistically significant.

## 3. Results

Mean age was 67.44 ± 5.11, median 69, interquartile range (IQR) 56 and 80 respectively. The female population count was 41, which accounts for 27.2%, while the male population was 110 (72.8%).

The Glasgow Coma Scale had a median of 13. A total of 73 patients had a GCS of 14 or 15, which accounts for nearly half of the patients (47.34%), while 42 patients had a GCS of 3–5, which accounts for 27.81%.

Hematoma thickness had a mean of 13.1 mm, with a standard deviation of 8.4 mm, with a median of 11 mm and IQR of 6–18 mm.

Midline shift (MLS) had a median of 5 mm and an IQR between 0 and 9.4 mm.

INR had a mean of 1.47 and a standard deviation of 0.89 and a median 1.18, with an IQR of 1.04–1.45. Exactly 42 patients (27.8%) had an INR above 1.4. A total of 23 patients had an INR above 2 (15.2%) while eight had an INR above 3 (5.2%).

**Figure 1:**
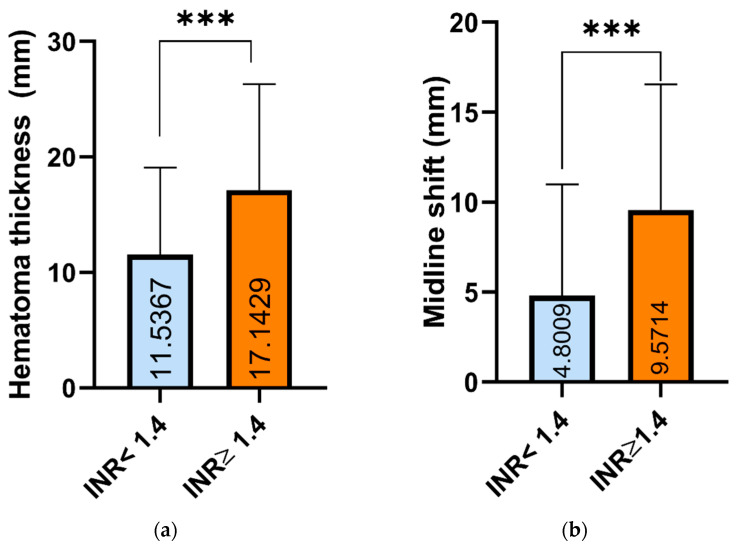
Comparative analysis of imaging parameters based on INR values: (**a**) average hematoma thickness in patients with INR < 1.4 compared to those with INR ≥ 1.4; (**b**) average MLS in patients with INR < 1.4 compared to those with INR ≥ 1.4. *** *p* < 0.001.

We noticed that the mean hematoma thickness in patients with an INR below 1.4 was 11.53, compared to 17.14 in patients with and INR above 1.4, as shown in Figure 1.

Furthermore, the mean MLS was 4.80 in patients with an INR below 1.4 and 9.57 in patients with INR above 1.4 drawn. These results are summarized in Figure 1.

Seven patients, totaling 4.6%, had pre-injury Eliquis.

Platelet count had a mean of 212.59 with a standard deviation of 91.18. The median was 206 with an IQR between 149 and 269.

Exactly 13 patients had a PLT of at most 100 (8.6%).

Neutrophile/Lymphocyte ratio (NLR) had a median of 5.93, with an IQR between 3.10 and 11.12.

A total of 88 patients underwent surgical intervention (58.3%).

Exactly 55 patients died while in hospital (36.4%).

The median length of hospital stay was 10 days, with an IQR between 6 and 17.

Due to non-normal distribution of variables assessed by Kolmogorov–Smirnov and Shapiro–Wilk normality tests, as well as our own visual inspection of data distribution on histograms, non-parametric statistical analysis was performed when using quantitative data.

Subsequently, univariate analysis was performed to identify the variables most significantly associated with the outcome of interest, as follows:

(a) The effect of age and sex on:

- Hematoma thickness and MLS.
- Admission GCS.
- Need for surgical intervention.
- Mortality.

These associations are summarized in Table 1 and Table 2.

Age was not significantly associated with hematoma thickness (*p* = 0.204), MLS (*p* = 0.583), admission GCS (*p* = 0.731), or need for surgical intervention (OR 1.006, 95% CI 0.985–1.028, *p* = 0.592). However, age was significantly associated with mortality (OR 1.03 per year, 95% CI 1.005–1.055, *p* = 0.016). Sex was not significantly associated with hematoma thickness (*p* = 0.560), MLS (*p* = 0.409), GCS (*p* = 0.250), mortality (OR 0.745, 95% CI 0.357–1.554, *p* = 0.435), or need for surgical intervention (OR 0.745, 95% CI 0.356–1.560, *p* = 0.435).

(b) The effect of INR on

- Hematoma thickness and MLS.
- GCS.
- Need for surgical intervention.
- Mortality.
- Length of hospital stay.

These associations are summarized in Table 3 and Table 4.

(c) The effect of hematoma thickness on

- MLS.
- GCS.
- Need for surgical intervention.
- Mortality.
- Length of hospital stay.

These associations are summarized in Table 5 and Table 6.

(d) The effect of GCS on

- Need for surgical intervention.
- Mortality.
- Length of hospital stay.

These associations are summarized in Table 7 and Table 8.

(e) The effect of NLR on

- Mortality.
- Length of hospital stay.

These associations are summarized in Table 9 and Table 10.

(f) The effect of platelet count on

- Hematoma thickness.
- MLS.
- GCS.
- Need for surgical intervention.
- Mortality.
- Length of hospital stay.

These associations are summarized in Table 11 and Table 12.

### 3.1. Multivariate Analysis

A multivariable logistic regression model including Glasgow Coma Scale (GCS), hematoma thickness (mm), elevated INR (with a cut-off value of ≥1.4), and age (years) was constructed based on clinical relevance and prior analyses. Predictors were selected based on a combination of clinical relevance and exploratory analyses. Initial univariate analyses identified Glasgow Coma Scale, INR, hematoma thickness, and age as candidate predictors.

A total of 151 patients were included in the analysis, of whom 55 (36.4%) died during hospitalization. All four variables included in the final model were complete and available for analysis, which provides an acceptable EPV (event-per-variable) ratio of 13.5. The results of the multivariate analysis are summarized in Table 13.

**Figure 2:**
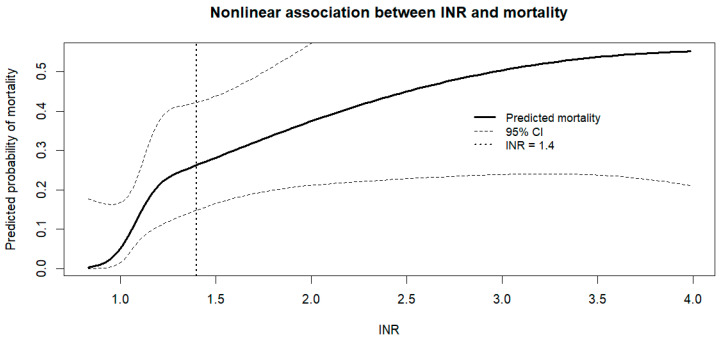
Nonlinear association between INR and mortality. Restricted spline modeling was used to evaluate the relationship between INR and predicted probability of in-hospital mortality, adjusted for GCS, hematoma thickness, and age. The curve demonstrates a nonlinear increase in mortality risk, with the steepest rise occurring around INR values of approximately 1.3–1.5. The vertical dashed line marks the prespecified, clinically interpretable threshold of INR ≥ 1.4, which was subsequently used in the final prediction model. This threshold was selected because the continuous INR model suggested nonlinearity and the spline curve identified INR ≈ 1.4 as lying within the main risk inflection zone. The plot was restricted to INR ≤ 4 to avoid unstable estimates at extreme INR values, where observations were sparse. Dashed curves represent 95% confidence intervals.

Elevated INR (≥1.4) was independently associated with mortality (OR 4.08, 95% CI 1.56–11.29; *p* = 0.005). The functional form of INR was further evaluated using spline modeling, which demonstrated a nonlinear association with mortality and supported dichotomization at a clinically interpretable threshold of ≥1.4. Final model specification was guided by clinical plausibility, avoidance of overfitting given the sample size, and confirmation of predictor stability using penalized regression. We initially attempted to perform the analysis using INR as a continuous variable. However, modeling INR as a continuous variable using restricted cubic splines demonstrated a significant nonlinear relationship with mortality, supporting the presence of a threshold effect, as shown in Figure 2. Based on this finding, INR was dichotomized at 1.4, which improved model calibration and interpretability.

Lower GCS was strongly associated with increased mortality (OR 1.27 per 1-point decrease, 95% CI 1.15–1.42; *p* < 0.001). Hematoma thickness (OR 1.07 per mm increase, 95% CI 1.00–1.14; *p* = 0.055) and age (OR 1.03 per year, 95% CI 1.00–1.07; *p* = 0.066) showed trends toward association after adjustment.

Multicollinearity diagnostics demonstrated no significant collinearity, with all variance inflation factors (VIFs) below 1.3.

### 3.2. Model Performance and Internal Validation

**Figure 3:**
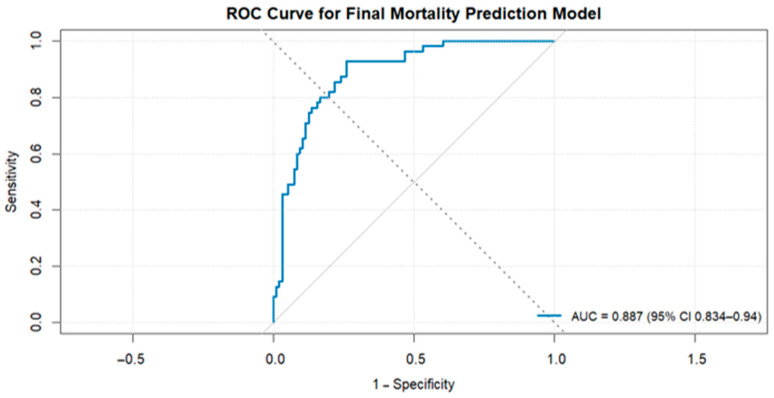
Receiver operating characteristics (ROC) curve for the mortality prediction model. The ROC curve demonstrates excellent discrimination of the final multivariable logistic regression model for in-hospital mortality, incorporating GCS, hematoma thickness, elevated INR (≥1.4), and age. The model achieved an AUC of 0.887 (95% CI 0.835–0.940—DeLong method), reflecting strong predictive performance. The gray lines and dash-lines represent reference lines. The light gray diagonal line represents the line of no discrimination (AUC = 0.5).

The model demonstrated very good discrimination, with an area under the receiver operating characteristic curve (AUC) of 0.8867 (0.834–0.9395), *p*-value < 0.001 (likelihood ratio test), as shown in Figure 3.

**Figure 4:**
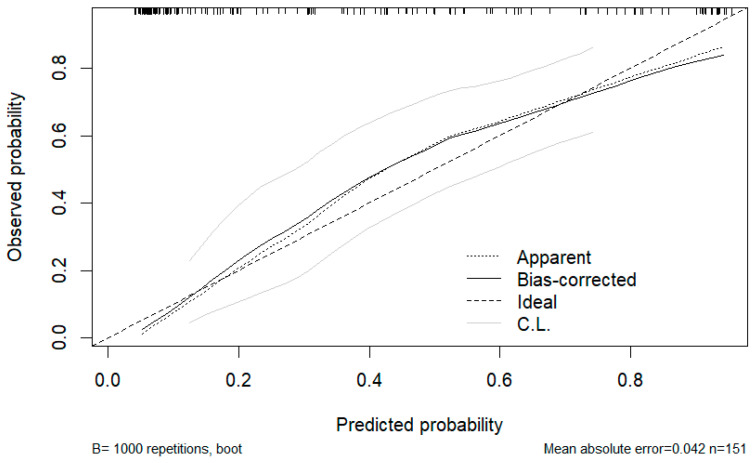
Calibration plot illustrating the agreement between predicted and observed probabilities of in-hospital mortality for the final multivariable logistic regression model, including Glasgow Coma Scale, hematoma thickness, elevated INR (≥1.4), and age. The apparent curve represents the model’s performance in the original dataset, while the bias-corrected curve was obtained using bootstrap resampling (1000 iterations). The dashed line indicates ideal calibration. The model demonstrated good calibration across the range of predicted probabilities, with minimal deviation from the ideal line, indicating accurate risk estimation.

Calibration analysis also showed good agreement between predicted and observed probabilities, with a calibration slope of 1.06 and no evidence of systematic miscalibration (intercept 0.02, *p* = 0.92). Overall predictive accuracy was high, with a Brier score of 0.132 (scaled Brier score 0.43), a mean absolute error of 0.034, and a mean squared error of 0.0018, indicating low prediction error across the full range of risk. The calibration plot is shown in Figure 4.

### 3.3. Decision Curve Analysis

**Figure 5:**
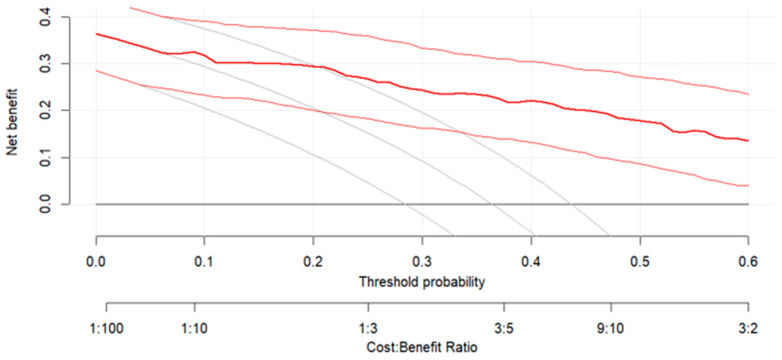
Decision curve analysis demonstrating the net clinical benefit of the final multivariable model for predicting in-hospital mortality across a range of threshold probabilities. The model, incorporating Glasgow Coma Scale, hematoma thickness, elevated INR (≥1.4), and age, consistently outperformed both the treat-all and treat-none strategies over clinically relevant threshold probabilities. The red solid line represents the model, while the gray lines correspond to the treat-all and treat-none approaches, respectively. Shaded areas indicate 95% confidence intervals derived from bootstrap resampling (1000 iterations). These findings support the clinical utility of the model for risk stratification and decision-making.

DCA (decision curve analysis) based on the final model demonstrated consistent net clinical benefit across clinically relevant threshold probabilities. Estimates were internally validated using bootstrap resampling (1000 iterations) to account for sampling variability. This supports the potential utility of the model for clinical risk stratification, as shown in Figure 5.

### 3.4. Penalized Regression and Model Stability

**Figure 6:**
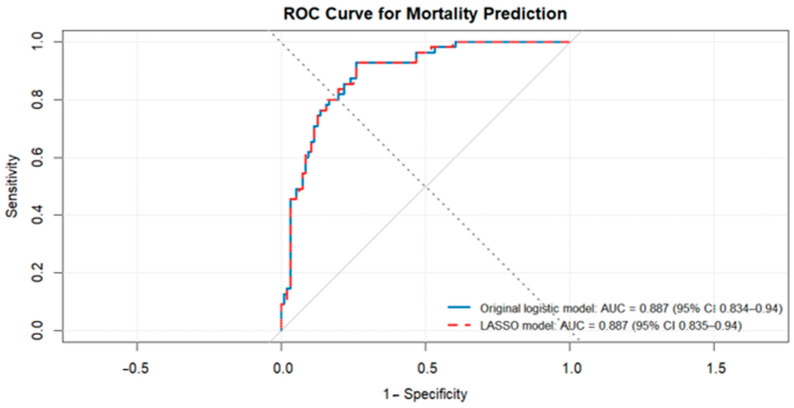
Receiver operating characteristic (ROC) curves comparing the discriminative performance of the standard multivariable logistic regression model and the LASSO-penalized model for predicting in-hospital mortality. Discrimination was excellent and nearly identical between approaches (original model AUC 0.8867 vs. LASSO AUC 0.8875). There was no significant difference between the two ROC curves (DeLong test *p* = 0.39), indicating that penalization did not materially improve model discrimination. The gray lines and dash-lines represent reference lines. The light gray diagonal line represents the line of no discrimination (AUC = 0.5).

A penalized logistic regression model using LASSO yielded similar predictive performance (AUC 0.8875, 95% CI: 0.8349–0.9401—DeLong method, *p*-value < 0.001—likelihood ratio test). Comparison of ROC curves using DeLong’s test showed no significant difference between the penalized and non-penalized models (Z = −0.85548, *p*-value = 0.3923), indicating that penalization did not materially improve discrimination. These findings suggest that the selected predictors form a stable and well-specified model with minimal risk of overfitting, as shown in Figure 6.

### 3.5. Mediation Analysis

**Figure 7:**
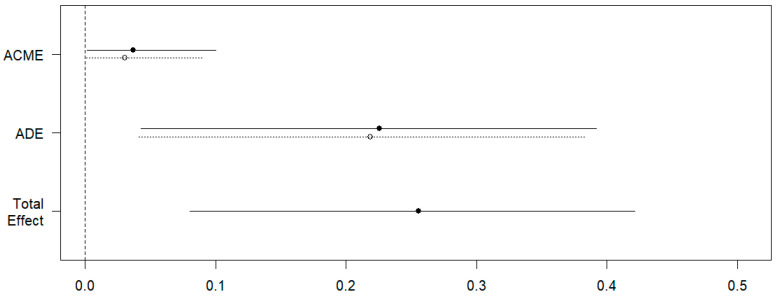
Causal mediation analysis illustrating the decomposition of the total effect of elevated INR (≥1.4) on in-hospital mortality into direct and indirect components mediated through hematoma thickness. The indirect effect (average causal mediation effect, ACME) was statistically significant but modest (estimate 0.034, 95% CI 0.001–0.095; *p* = 0.028), accounting for approximately 13% of the total effect. The direct effect (average direct effect, ADE) remained substantial and significant (estimate 0.222, 95% CI 0.042–0.386; *p* = 0.016). These findings indicate that hematoma thickness represents a partial mediating pathway linking coagulopathy to mortality, while most of the effect is mediated through additional mechanisms. Error bars represent 95% bootstrap confidence intervals (1000 simulations). The filled dots represent the estimated effects, while the empty dots represent bias-corrected effects. Horizontal lines represent the corresponding 95% bootstrap confidence intervals, and the vertical dashed line indicates the null effect (effect = 0).

Causal mediation analysis was performed to explore whether hematoma thickness mediates the relationship between coagulopathy and mortality. Elevated INR was significantly associated with increased mortality (total effect 0.256, 95% CI 0.080–0.421; *p* = 0.002). The indirect effect mediated through hematoma thickness was statistically significant but modest (ACME 0.034, 95% CI 0.001–0.095; *p* = 0.028), accounting for approximately 13% of the total effect. The direct effect remained substantial and statistically significant (ADE 0.222, 95% CI 0.042–0.386; *p* = 0.016), as shown in Figure 7.

These findings indicate that hematoma burden represents a partial mediating pathway linking coagulopathy to mortality, while the majority of the effect of INR appears to be mediated through additional mechanisms.

## 4. Discussion

In this study, the analyzed cohort reflects a predominantly elderly population, with a mean age of 67.4, a relatively narrow standard deviation (+/−5.11), and a slightly wider interquartile range of 56–80. This distribution shows a skew toward an increased age, which is consistent with the epidemiology of aSDH in the literature [9,10,11]. Age was a modest but significant predictor of mortality, with each year of age increasing the mortality rate by 1.03 times, which might be explained by the physiological frailty and reduced functional reserve of this population group, rather than the severity of the primary injury itself. As such, univariate analysis showed that age was not associated with any imaging marker of severity (hematoma thickness or midline shift), nor with the GCS or the need for surgical intervention.

The cohort was predominantly male, which is consistent with known trauma epidemiology [12,13]. However, sex did not influence any of the analyzed outcome parameters, thus demonstrating a limited prognostic value in this cohort. As far as the neurological status is concerned, the GCS presented a bimodal distribution, with a large proportion of patients in the “mild-TBI” subgroup (GCS 13–15) and a smaller but considerable subgroup in the “severe-TBI” category (GCS 3–8, especially the 3–5 interval). This distribution is reflected in outcome variability and underscores the importance of GCS as a key outcome predictor, which will be further elaborated in this section. Additionally, GCS proved to be the most powerful clinical predictor, strongly associated with mortality, surgical intervention and length of hospital stay, which is consistent with existing literature data [14,15,16,17].

As far as imaging parameters are concerned, hematoma thickness (mean 13.1 mm, median 11 mm) and midline shift (median 5 mm) demonstrated large variability, indicating a broad spectrum of mass effect within the cohort. Among these two, hematoma thickness emerged as the strongest structural determinant, demonstrating a very strong association with midline shift (R^2^ = 0.661, *p* < 0.001) and a low-moderate but significant association with GCS, need for surgical intervention and mortality. These findings suggest that hematoma thickness is a central driver of both mechanical brain compression, secondary injury and downstream clinical deterioration, as well as a major factor guiding surgical decision-making.

From a biological perspective, coagulation status played a major role. INR (mean 1.47, with 15.2% patients above 2 and 5.2% above 3) was significantly associated with hematoma thickness, midline shift, GCS and mortality, highlighting its contribution to both bleeding severity and clinical outcome.

In our cohort, INR was not significantly associated with the decision of surgical intervention. However, the discussion regarding the influence of INR on the surgical decision is complex and might appear paradoxical. On the one hand, surgeons often hesitate to operate on patients with increased INR, which is being perceived as a relative contraindication for surgery, due to the increased intraoperative bleeding risk and perioperative complications associated with hemostatic disturbances. On the other hand, our analysis demonstrated that higher INR values are significantly associated with increased hematoma thickness and midline shift. These parameters represent the primary drivers of surgical decision-making in aSDH. In other words, by increasing the hematoma thickness and midline shift, INR increases the likelihood that an aSDH would require surgical intervention. This apparent contradiction can be explained by the distinction between direct and indirect effects. While coagulopathy may promote hematoma expansion and thereby increase radiological severity, the ultimate decision to operate appears to be determined by structural and clinical factors rather than by INR itself. In this context, INR may act as an upstream modifier of disease severity rather than an independent determinant of surgical management.

Platelet count showed a similar but weaker pattern, with lower values associated with increased hematoma thickness, mortality and surgical intervention, although effect sizes were small, indicating a secondary role, compared to INR.

Inflammatory status was assessed using the Neutrophile-to-Lymphocyte ratio (NLR). This index was associated with both mortality and length of hospital stay, but with modest explanatory power. This suggests that, although systemic inflammation is not a determinant of the initial injury severity, it contributes to outcome and recovery. Nevertheless, the length of hospital stay (median of 10 days) appeared more closely related to admission GCS, rather than structural, coagulation, or systemic inflammation parameters.

Overall, these findings highlight a hierarchical model of disease severity, in which GCS emerges as the strongest predictor of mortality. Hematoma thickness represents the main structural parameter, influencing mass effect, neurological status and the need for surgical intervention. Biological factors, such as INR, platelet count and systemic inflammation (assessed by NLR), also appear to influence outcomes, particularly mortality, though with a smaller impact than GCS or hematoma thickness. Although age has been associated with mortality, it does not influence initial injury characteristics, suggesting that this correlation is rather attributable to the decreased physiological resilience of patients in this population subgroup.

### 4.1. Multivariate Analysis

We developed a multivariable model including age, GCS, hematoma thickness and INR as predictors of mortality. This model was internally validated, while showing very good discrimination (AUC ~0.89), good calibration, and consistent clinical utility, suggesting that a limited set of readily available clinical variables can provide accurate early risk stratification in patients with subdural hematoma.

Because surgical intervention represents a major therapeutic decision that may influence outcome, we performed an additional sensitivity analysis including surgical intervention in the multivariable model. Although surgical intervention was strongly associated with mortality in univariate analysis (OR 7.88, *p* < 0.001), this association was no longer statistically significant after adjustment for Glasgow Coma Scale score, hematoma thickness, age, and INR (OR 2.10, 95% CI 0.659–6.822; *p* = 0.209). In contrast, elevated INR remained independently associated with in-hospital mortality (OR 3.86, 95% CI 1.47–10.75; *p* = 0.007), while overall model discrimination remained excellent (AUC 0.891, 95% CI 0.840–0.941). These findings suggest that the increased mortality observed among surgically treated patients is primarily attributable to their greater baseline clinical and radiological severity rather than to an independent adverse effect of the intervention itself. Indeed, surgical intervention is generally reserved for patients presenting with more severe neurological impairment and larger hematomas, factors that are themselves strongly associated with mortality. Therefore, the apparent relationship between surgical intervention and mortality appears to reflect underlying disease severity rather than a direct negative effect of surgical management.

Although age was not statistically significant in the fully adjusted model, it was kept in the multivariate model due to its consistent association with mortality in prior analyses and the broader literature [18,19,20], as well as strong clinical plausibility. The attenuation of statistical significance after adjustment likely reflects shared variance with other predictors (particularly neurological status and hematoma burden) and limited power rather than the absence of a true effect. Importantly, inclusion of age improved overall model performance. This was reflected by better calibration, a lower Brier score, and increased clinical utility on decision curve analysis, supporting its contribution to risk estimation at the model level. In order to avoid the bias and instability associated with data-driven variable selection, predictors were selected based on clinical relevance and overall predictive performance rather than statistical significance alone. Excluding age resulted in subtle but consistent deterioration in calibration and overall prediction error, further supporting its inclusion.

These findings suggest that mortality appears to be primarily driven by neurological injury, assessed using GCS, as well as systemic vulnerability. Coagulopathy seems to act both directly and indirectly, through hematoma expansion. As such, only ~13% of the INR effect is mediated through hematoma thickness, which may imply that the prognostic effect of coagulopathy extends beyond radiological hemorrhage burden alone and may also reflect systemic vulnerability, ongoing bleeding tendency, perioperative complications, or diffuse secondary injury mechanisms. The nonlinear relationship between INR and mortality, demonstrated by spline modeling, supports the presence of a risk inflection zone rather than a linear effect, justifying the use of a clinically interpretable threshold. The threshold of 1.4 lies within the steepest segment of the risk curve, suggesting that relatively small increases in coagulopathy beyond this point are associated with disproportionate increases in mortality risk. The INR threshold of 1.4 warrants further discussion. In neurosurgical practice, INR values above 1.4 are commonly considered indicative of the need for coagulation reversal therapy prior to surgical intervention, as suggested by neurotrauma management guidelines [21]. Consequently, anticoagulated patients may experience delays in receiving life-saving surgical evacuation compared to non-coagulopathic patients. Several studies have shown that delayed surgical intervention in traumatic aSDH is associated with increased mortality, with some authors suggesting an optimal intervention window of approximately 4 h following trauma [7]. As such, the increased mortality observed in patients with INR values greater than 1.4 may not only reflect the biological effects of coagulopathy itself, but also the consequences of delayed surgical management. In this regard, a cohort study published in The Journal of Trauma and Acute Care Surgery suggested that moderately elevated INR values should not systematically delay neurosurgical intervention following TBI [21].

The model demonstrated strong ability to distinguish between survivors and non-survivors, while the close agreement between predicted and observed risk supports good reliability for individual risk estimation in this cohort. The consistent net benefit across clinically relevant thresholds suggests that the model may support decision-making rather than merely risk prediction. However, further multicenter external validation of the proposed prediction model is necessary before broader clinical implementation.

Nevertheless, the absence of substantial improvement with penalization suggests reasonable model stability and limited evidence of major overfitting within this cohort.

### 4.2. Limitations

This study has several limitations. First, due to its retrospective observational design, clear causal relationships cannot be established; whereas, the possibility of residual confounding still remains, even after adjustment for clinically relevant variables. Particularly, the mediation analysis assumes the absence of unmeasured confounding between exposure, mediator, and outcome variables, an assumption that cannot be fully guaranteed in retrospective studies and may have influenced the estimated indirect effects. The impact of INR may also be caused by individual surgical decisions and not necessarily by its biological implications. The etiology of elevated INR could not be reliably determined in all patients. Although information regarding anticoagulant use was available for a subset of cases, other potential causes of INR elevation, including trauma-induced coagulopathy and underlying systemic disorders affecting hemostasis, were not consistently documented. Therefore, we were unable to assess whether the prognostic impact of elevated INR varied according to its underlying mechanism.

Secondly, the exclusion of patients with bilateral hematomas, associated cerebral contusions or epidural hematomas, which are frequently encountered in neurotrauma emergency settings, may also represent a limitation. While our aim was to create a more homogenous study population and reduce confounding, excluding these patients could mean that the present findings may not be fully generalizable to the broader population of patients with complex traumatic intracranial hemorrhagic lesions, making them primarily interpretable in the context of relatively isolated aSDH. Moreover, the sample size, although adequate for model development (EPV ~13.5), remains modest and may limit the precision of effect estimates, particularly for secondary analyses such as mediation, where confidence intervals were relatively wide.

Thirdly, the model was only internally validated using bootstrap resampling, lacking external validation, which is necessary to confirm generalizability to other populations and clinical settings. Additionally, hematoma thickness was only used as a static parameter without taking into consideration certain dynamic features such as hematoma expansion, rate of bleeding, or temporal evolution, which may partly explain the limited proportion of the INR effect mediated through this variable. Information regarding time from admission (or injury) to surgical intervention was unavailable for most patients and therefore could not be incorporated into the analyses. Consequently, we were unable to evaluate whether delays related to coagulation reversal or surgical preparation contributed to in-hospital mortality. Dichotomization of INR, while supported by nonlinear modeling and improved calibration, may result in some loss of information and may not fully capture the continuous nature of coagulopathy. Potential measurement variability in imaging parameters and clinical assessment (e.g., GCS) may also introduce non-differential misclassification.

Furthermore, although mediation analysis suggested that hematoma thickness may act as an intermediary variable linking elevated INR and mortality, both variables were measured at admission and therefore the temporal assumptions required for causal mediation cannot be fully verified. Accordingly, these findings should be interpreted as exploratory and require confirmation in prospective studies incorporating serial imaging and longitudinal assessment of coagulation status.

Finally, the retrospective nature of the study introduces inherent methodological limitations, including the potential for selection bias, incomplete documentation, heterogeneity in treatment strategies, and inability to control for all potentially relevant confounding variables; retrospective data collection relies on the accuracy and completeness of medical records and imaging archives, which may introduce measurement variability and information bias despite standardized institutional protocols.

### 4.3. Future Perspectives

Further research could be useful in evaluating the potential role of early prehospital detection and correction of coagulopathy in patients with acute head trauma. Rapid assessment of coagulation status at the scene of injury or during transport to the hospital could potentially reduce delays in surgical management and allow earlier initiation of reversal therapy in selected patients. The increasing availability of portable point-of-care coagulation testing devices may facilitate rapid identification of high-risk patients in emergency settings. As such, future prospective studies are needed to determine whether prehospital coagulation-guided management may improve neurological outcome and survival.

## 5. Conclusions

Traumatic aSDH outcome appears to be determined by an interaction between neurological severity, structural hemorrhage burden, and biological vulnerability. Admission GCS remained the strongest predictor of mortality in our cohort, while coagulopathy, particularly INR ≥ 1.4, was independently associated with poor outcome even after adjustment for radiological severity markers. These findings support the potential prognostic relevance of coagulation abnormalities in traumatic aSDH and suggest that integration of clinical, radiological, and coagulation parameters may aid risk stratification in this patient population. However, these observations should be considered exploratory and require confirmation in larger multicenter studies with external validation.

## Figures and Tables

**Table 1:** Univariate regression analysis evaluating the association between age and hematoma thickness, MLS, admission Glasgow Coma Scale (GCS) score, need for surgical intervention, and mortality.

Outcome	B	SE	β	*p*-Value	95% CI (Lower–Upper)	R^2^
Hematoma thickness	0.058	0.045	0.104	0.204	−0.032–0.147	0.011
Midline shift	0.020	0.037	0.045	0.583	−0.052–0.092	0.002
GCS	0.009	0.026	0.028	0.731	−0.043–0.061	0.001
Mortality	0.029	0.012	—	0.016	1.005–1.055 ‡	—
Intervention	0.006	0.011	—	0.592	0.985–1.028 ‡	—

‡ Confidence intervals are reported for odds ratios because regression coefficients (β) are not displayed for logistic regression outcomes.

**Table 2:** Univariate logistic regression analysis evaluating the effect of age on mortality and need for surgical intervention in patients with traumatic aSDH.

Outcome	OR (Exp B)	95% CI	Model *p*-Value	Nagelkerke R^2^
Mortality	1.030	1.005–1.055	0.013	—
Intervention	1.006	0.985–1.028	0.592	0.003

**Table 3:** Univariate regression analyses evaluating the association between admission INR and clinical, radiological, and outcome variables in patients with traumatic aSDH.

Outcome	B	SE	β	*p*-Value	95% CI (Lower–Upper)	R^2^
Hematoma thickness	2.754	0.739	0.292	<0.001	1.295–4.213	0.085
Midline shift	2.456	0.587	0.324	<0.001	1.296–3.616	0.105
GCS	−0.890	0.440	−0.163	0.045	−1.760–−0.020	0.027
Length of stay (days)	0.190	1.242	0.013	0.879	−2.264–2.643	0.000
Mortality	1.064	0.321	—	0.001	1.544–5.435 ‡	—
Surgical intervention	0.274	0.221	—	0.214	0.853–2.028 ‡	—

‡ Confidence intervals are reported for odds ratios because regression coefficients (β) are not displayed for logistic regression outcomes.

**Table 4:** Univariate logistic regression analysis evaluating the effect of INR on mortality and need for surgical intervention in patients with traumatic aSDH.

Outcome	OR (Exp B)	95% CI	Model *p*-Value	Nagelkerke R^2^
Mortality	2.897	1.544–5.435	<0.001	0.153
Intervention	1.316	0.853–2.028	0.182	0.016

INR was significantly associated with hematoma thickness, MLS (*p* < 0.001), GCS (*p* = 0.04), and mortality (*p* = 0.001), but not with surgical intervention or length of hospital stay (*p* = 0.879).

**Table 5:** Univariate regression analyses evaluating the association between hematoma thickness and clinical, radiological, and outcome variables in patients with traumatic aSDH.

Outcome	B	SE	β	*p*-Value	95% CI (Lower–Upper)	R^2^
Midline shift	0.653	0.038	0.813	<0.001	0.578–0.729	0.661
GCS	−0.306	0.040	−0.529	<0.001	−0.385–−0.227	0.280
Length of stay (days)	0.131	0.131	0.082	0.320	−0.128–0.391	0.007
Mortality	0.144	0.028	—	<0.001	1.092–1.221 ‡	—
Surgical intervention	0.315	0.053	—	<0.001	1.234–1.521 ‡	—

‡ Confidence intervals are reported for odds ratios because regression coefficients (β) are not displayed for logistic regression outcomes.

**Table 6:** Univariate logistic regression analysis evaluating the effect of hematoma thickness on mortality and need for surgical intervention in patients with traumatic aSDH.

Outcome	OR (Exp B)	95% CI	Model *p*-Value	Nagelkerke R^2^
Mortality	1.155	1.092–1.221	<0.001	0.285
Intervention	1.370	1.234–1.521	<0.001	—

Hematoma thickness was strongly associated with MLS (R^2^ = 0.661, *p* < 0.001), GCS (R^2^ = 0.280, *p* < 0.001), mortality (OR 1.155 per mm, *p* < 0.001), and need for surgical intervention (OR 1.37 per mm, *p* < 0.001). No significant association was observed with length of hospital stay (*p* = 0.320).

**Table 7:** Univariate regression analyses evaluating the association of GCS and clinical outcome variables in patients with traumatic aSDH.

Outcome	B	SE	β	*p*-Value	95% CI (Lower–Upper)	R^2^
Length of stay (days)	−0.656	0.222	−0.236	0.004	−1.094–−0.218	0.056
Mortality	−0.261	0.042	—	<0.001	0.709–0.837 ‡	—
Surgical intervention	−0.284	0.053	—	<0.001	0.679–0.834 ‡	—

‡ Confidence intervals are reported for odds ratios because regression coefficients (β) are not displayed for logistic regression outcomes.

**Table 8:** Univariate logistic regression analysis evaluating the effect of GCS on mortality and need for surgical intervention in patients with traumatic aSDH.

Outcome	OR (Exp B)	95% CI	Model *p*-Value	Nagelkerke R^2^	Hosmer–Lemeshow
Mortality	0.770	0.709–0.837	<0.001	0.368	0.431 (good)
Intervention	0.752	0.679–0.834	<0.001	0.347	0.019 (poor)

GCS was strongly associated with mortality (OR 0.77 per point, 95% CI 0.71–0.84, *p* < 0.001) and need for surgical intervention (OR 0.75, 95% CI 0.68–0.83, *p* < 0.001). Lower GCS was also associated with longer hospital stay (B = −0.66, *p* = 0.004).

**Table 9:** Univariate regression analyses evaluating the association of NLR and clinical outcome variables in patients with traumatic aSDH.

Outcome	B	SE	β	*p*-Value	95% CI (Lower–Upper)	R^2^
Length of stay (days)	0.256	0.116	0.178	0.029	0.027–0.484	0.032
Mortality	0.057	0.023	—	0.012	1.013–1.107 ‡	—

‡ Confidence intervals are reported for odds ratios because regression coefficients (β) are not displayed for logistic regression outcomes.

**Table 10:** Univariate logistic regression analysis evaluating the effect of NLR on mortality in patients with traumatic aSDH.

Outcome	OR (Exp B)	95% CI	Model *p*-Value	Nagelkerke R^2^	Hosmer–Lemeshow
Mortality	1.059	1.013–1.107	0.004	0.073	0.161 (good fit)

NLR was associated with both mortality (OR 1.059 per unit, 95% CI 1.013–1.107, *p* = 0.012) and length of hospital stay (B = 0.256, *p* = 0.029); although, the effect sizes and explanatory power were modest.

**Table 11:** Univariate regression analyses evaluating the association between platelet count (PLT) and clinical, radiological, and outcome variables in patients with traumatic aSDH.

Outcome	B	SE	β	*p*-Value	95% CI (Lower–Upper)	R^2^
Hematoma thickness	−0.015	0.007	−0.168	0.039	−0.030–−0.001	0.028
Midline shift	−0.007	0.006	−0.100	0.221	−0.019–0.005	0.010
GCS	0.005	0.004	0.092	0.259	−0.004–0.013	0.009
Mortality	−0.006	0.002	—	0.004	0.989–0.998 ‡	—
Surgical intervention	−0.004	0.002	—	0.026	0.992–0.999 ‡	—

‡ Confidence intervals are reported for odds ratios because regression coefficients (β) are not displayed for logistic regression outcomes.

**Table 12:** Univariate logistic regression analysis evaluating the effect of PLT on mortality and need for surgical intervention in patients with traumatic aSDH.

Outcome	OR (Exp B)	95% CI	Model *p*-Value	Nagelkerke R^2^	Hosmer–Lemeshow
Mortality	0.994	0.989–0.998	0.002	0.084	0.033 (poor)
Intervention	0.996	0.992–0.999	0.022	—	—

Lower platelet counts were associated with increased hematoma thickness (B = −0.015, *p* = 0.039), higher mortality (OR 0.994 per unit, 95% CI 0.989–0.998, *p* = 0.004), and increased likelihood of surgical intervention (OR 0.996, *p* = 0.026); although, effect sizes were small.

**Table 13:** Multivariate logistic regression analysis evaluating independent predictors of mortality in patients with traumatic aSDH.

Variable	β Coefficient	SE (Standard Error)	OR (95% CI)	*p*-Value
GCS (per 1-point decrease)	0.241	0.053	1.27 (1.15–1.42)	<0.001
Hematoma thickness (per mm increase)	0.064	0.033	1.07 (1.00–1.14)	0.055
INR ≥ 1.4 (yes vs. no)	1.406	0.501	4.08 (1.56–11.29)	0.005
Age (per 1-year increase)	0.033	0.018	1.03 (1.00–1.07)	0.066

Hosmer–Lemeshow test—0.126 (good fit).

## Data Availability

The data presented in this study are available on request from the corresponding author due to privacy restrictions related to patient confidentiality.

## Abbreviations

**aSDH**—acute subdural hematoma; **ADE**—average direct effect; **ACME**—average causal mediation effect; **AUC**—area under the receiver operating characteristic curve; **CI**—confidence interval; **CT**—computed tomography; **DCA**—decision curve analysis; **EPV**—events per variable; **GCS**—Glasgow Coma Scale; **ICD-10**—International Classification of Diseases, Tenth Revision; **INR**—international normalized ratio; **IQR**—interquartile range; **LASSO**—least absolute shrinkage and selection operator; **LMIC**—low- and middle-income country; **MLS**—midline shift; **NLR**—Neutrophile-to-Lymphocyte ratio; **OR**—odds ratio; **PLT**—platelet count; **ROC**—receiver operating characteristic; **SPSS**—Statistical Package for the Social Sciences; **TBI**—traumatic brain injury; **VIF**—variance inflation factor.
